# Mediterranean diet, Mindfulness-Based Stress Reduction and usual care during pregnancy for reducing fetal growth restriction and adverse perinatal outcomes: IMPACT BCN (*Improving Mothers for a better PrenAtal Care Trial BarCeloNa*): a study protocol for a randomized controlled trial

**DOI:** 10.1186/s13063-021-05309-2

**Published:** 2021-05-24

**Authors:** Francesca Crovetto, Fàtima Crispi, Roger Borras, Cristina Paules, Rosa Casas, Andrés Martín-Asuero, Angela Arranz, Eduard Vieta, Ramon Estruch, Eduard Gratacós

**Affiliations:** 1 BCNatal | Fetal Medicine Research Center (Hospital Clínic and Hospital Sant Joan de Déu), Institut d’Investigacions Biomèdiques August Pi i Sunyer (IDIBAPS), University of Barcelona, Barcelona, Spain; 2 Centre for Biomedical Research on Rare Diseases (CIBER-ER), Madrid, Spain; 3 Cardiovascular Institute, Hospital Clínic, IDIBAPS, University of Barcelona, Barcelona, Spain; 4 Centro de Investigación Biomédica en Red de Fisiopatología de la Obesidad y Nutrición (CIBERON), Instituto de Salud Carlos III, Madrid, Spain; 5 Instituto esMindfulness, Barcelona, Spain; 6 Department of Psychiatry and Psychology, Hospital Clinic, Neuroscience Institute, IDIBAPS, CIBERSAM, University of Barcelona, Barcelona, Spain; 7 Department of Internal Medicine Hospital Clinic, IDIBAPS, University of Barcelona, Barcelona, Spain

**Keywords:** Pregnancy, Fetal growth restriction, Mediterranean diet, Mindfulness-Based Stress Reduction, Perinatal outcome

## Abstract

**Background:**

Fetal growth restriction (FGR) affects 7–10% of all pregnancies resulting in a higher risk of perinatal morbidity and mortality, long-term disabilities, and cognitive impairment. Due to its multifactorial etiology, changes in maternal lifestyle, including suboptimal maternal diet and stress, have increasingly been associated with its prevalence. We present a protocol for the *Improving Mothers for a better PrenAtal Care Trial Barcelona* (IMPACT BCN), which evaluates two different maternal lifestyle strategies (improved nutrition by promoting Mediterranean diet and stress reduction program based on mindfulness techniques) on perinatal outcomes. The primary objective is to reduce the prevalence of FGR. Secondary aims are to reduce adverse perinatal outcomes and to improve neurodevelopment and cardiovascular profile in children at 2 years of age.

**Methods:**

A randomized parallel, open-blind, single-center trial following a 1:1:1 ratio will select and randomize high-risk singleton pregnancies for FGR (*N*=1218), according to the criteria of the Royal College of Obstetricians and Gynaecologists (19.0–23.6 weeks’ gestation), into three arms: Mediterranean diet, mindfulness-based stress reduction program, and usual care without any intervention. Compliance to the interventions will be randomly tested in 30% of participants with specific biomarkers. Maternal socio-demographic, clinical data, biological samples, and lifestyle questionnaires will be collected at enrollment and at the end of the interventions (34.0–36.6 weeks’ gestation), together with a fetoplacental ultrasound and magnetic resonance. Fetoplacental biological samples and perinatal outcomes will be recorded at delivery. Postnatal follow-up is planned up to 2 years of corrected age including neurodevelopmental tests and cardiovascular assessment. Intention-to-treat and population per-protocol analysis will be performed.

**Discussion:**

This is the first randomized study evaluating the impact of maternal lifestyle interventions during pregnancy on perinatal outcomes. The maternal lifestyle interventions (Mediterranean diet and mindfulness-based stress reduction program) are supported by scientific evidence, and their compliance will be evaluated with several biomarkers.

**Trial registration:**

ClinicalTrials.govNCT03166332. Registered on April 19, 2017

## Administrative information

**Table:** 

Title {1}	Mediterranean Diet, Mindfulness Based Stress Reduction and usual care during pregnancy for reducing fetal growth restriction and adverse perinatal outcomes: IMPACT BCN (*Improving Mothers for a better PrenAtal Care Trial BarCeloNa*). A study protocol for a randomized controlled trial.
Trial registration {2a and 2b}.	ClinicalTrials.gov, NCT03166332. Registered 19 April 2017 - Prospectively registered before any outcome occurs. https://clinicaltrials.gov/ct2/results?cond=&term=NCT03166332&cntry=&state=&city=&dist=.
Protocol version {3}	Version 2, 23.10.2019
Funding {4}	The project is funded by: a grant from “La Caixa” Foundation (LCF/PR/GN18/10310003); Cerebra Foundation for the Brain Injured Child (Carmarthen, Wales, UK), and AGAUR under grant 2017 SGR n° 1531. Additionally, F. Crovetto has received funding from Centro de Investigaciones Biomédicas en Red sobre Enfermedades Raras (CIBERER).
Author details {5a}	Francesca Crovetto^1,2^, MD, PhD; Fàtima Crispi^1,2^, MD, PhD; Roger Borras^1,3^, PhD; Cristina Paules^1^, MD, PhD; Rosa Casas^4^, PhD; Andrés Martín^5^, PhD; Angela Arranz^1^, PhD; Eduard Vieta^6^, MD, PhD; Ramon Estruch^4,7^, MD, PhD; Eduard Gratacos^1,2^, MD, PhD. ^1^BCNatal | Fetal Medicine Research Center (Hospital Clínic and Hospital Sant Joan de Déu), University of Barcelona, Institut d’Investigacions Biomèdiques August Pi i Sunyer (IDIBAPS), Barcelona, Spain ^2^Centre for Biomedical Research on Rare Diseases (CIBER-ER), Madrid, Spain ^3^Cardiovascular Institute, Hospital Clínic, University of Barcelona, Barcelona, Spain; ^4^Centro de Investigación Biomédica en Red de Fisiopatología de la Obesidad y Nutrición (CIBERON), Instituto de Salud Carlos III, Madrid, Spain; ^5^Instituto esMindfulness, Barcelona, Spain; ^6^Department of Psychiatry and Psychology, Hospital Clinic, Neuroscience Institute, IDIBAPS, University of Barcelona, CIBERSAM, Barcelona, Spain; ^7^Department of Internal Medicine Hospital Clinic, IDIBAPS, University of Barcelona, Barcelona, Spain.
Name and contact information for the trial sponsor {5b}	Several founders, the principal one is La Caixa Foundation. Contact: healthresearcher@fundaciolacaixa.org
Role of sponsor {5c}	The funders had no role in the study design; data collection, data analysis, data interpretation; or writing.

## Introduction

### Background and rationale {6a}

Fetal growth restriction (FGR) is defined as the failure to achieve the endorsed growth potential in utero, and it affects 7–10% of all pregnancies [1]. FGR is usually related to placental insufficiency and may present in association with other prevalent complications of pregnancy, mainly prematurity and preeclampsia [2]. The detection of small fetuses is clinically relevant, because it identifies a subgroup of pregnancies at high risk of poorer perinatal outcomes, such as intrauterine fetal death, severe intrapartum fetal distress, and perinatal brain injury [3, 4]. In addition, children born small have poorer neurobehavioral competencies [5] and more behavioral, sensory, and cognitive dysfunctions later in life [6, 7]. Being born small also expose them to a higher risk for subclinical cardiac dysfunction and hypertension in childhood [8] and a higher predisposition to cardiovascular disease in adult life [9]. While placental insufficiency seems a common feature in most cases of FGR, it is accepted that the etiology of FGR is multifactorial. Recent research findings point at suboptimal maternal diet [10–12] and maternal stress [13, 14] (Miranda J, Macias-Redondo S, Paules C, Gomez ACF, et al: Maternal stress and placental RNA expression and methylation of the HSD11β2 gene in fetal growth restriction, submitted) as potentially important contributors.

#### Nutrition during pregnancy

The role of maternal and fetal nutrition is well accepted to have a profound impact not only on the early life growth and development, but also on the lifelong health of the individual [15]. Maternal intake of several micronutrients related with fetal growth [16] has been reported to be commonly suboptimal in women from developed countries [17]. In a recent comprehensive metabolomic study [11], we observed alterations in the lipid profiles of FGR fetuses and their mothers. Specifically, mothers of FGR fetuses showed a low concentration of cholesterol and other lipoproteins, which might reflect increased uptake into the placenta and explain the reported increase in circulating triglycerides and cholesterol lipoproteins in growth-restricted fetuses.

Dietary pattern is a key factor in the prevention of several chronic diseases [18]. Concerning pregnancy, rather than individual constituents of food in the form of supplemental intakes of specific micronutrients [19–22], a dietary pattern as a whole may have a great role in achieving optimal maternal nutrition during pregnancy [23]. In this context, the Mediterranean diet (MedDiet), rich in mono- and polyunsaturated fatty acids [24], is an attractive option. Known to reduce the incidence of cardiovascular diseases in adults [18], the MedDiet could as well improve pregnancy outcomes and fetal growth. For instance, in a trial assessing the impact of diet on macrosomia, low adherence to the MedDiet was associated with low birth weight (BW) [25]. In pregnant women from the Generation R study in the Netherlands, both low and medium adherence to the MedDiet was associated with lower BW [10]. In two population-based cohort studies from Spain, higher intakes of fish, legumes, and dairy products were associated with higher infant BW and lower risk of FGR [12]. Similarly, in women from the Danish National Birth Cohort, higher intake of Mediterranean foods reduced the risk for preterm birth [26]. However, all these studies were observational. Therefore, randomized interventional studies powered to test the effect of MedDiet on fetal growth and perinatal outcomes are needed.

#### Stress during pregnancy

The association between maternal stress and FGR has long been described [27]. An over-exposure to stress-induced maternal glucocorticoids has been proposed as a potential mediating mechanism [13, 28]. Evidence in rat models showed that maternal prenatal stress can downregulate the placental enzyme 11β-hydroxysteroid dehydrogenase type 2 (11β-HSD2) [29], essential to oxidize cortisol into cortisone. Recent studies in humans also found direct evidence that maternal prenatal anxiety and depression are associated with a downregulation of the 11β-HSD2 gene expression [30, 31]. Analyses on cord blood samples have revealed higher cortisol and lower adrenocorticotropic hormone levels in cases of FGR [27].

In recent years, accumulating evidence supports the notion that adverse events or external negative stimulus during pregnancy affect maternal psychological well-being and have the potential to alter placental physiology with deleterious consequences in fetal development [14, 32]. In order to reduce stress in pregnancy, a non-pharmacological intervention would be desirable. Mindfulness-Based Stress Reduction (MBSR) is a promising tool for treating anxiety and mood problems in patients with various clinical conditions [33]. There are few published studies on the use of MBRS programs in pregnancy. A recent meta-analysis pooling the results of randomized controlled trials (RCTs) [34] reported a non-significant trend in favor of MBRS interventions to reduce anxiety, depression, and perceived stress. This may be due to a small number of RCTs and participants. The RCTs available were either pilot or feasibility studies, or not adequately powered to demonstrate the effectiveness of a MBSR intervention. A small study suggested that mindfulness-related skills were associated with a reduced chance of delivering a low-BW newborn [35]. Thus, more robust evidence is needed on the impact of MBSR strategies on perinatal outcomes.

#### Justification for the study

Maternal psychological status and dietary pattern are both fundamental for pregnancy well-being and contribute to shape the growth and development of the fetus and future child [36]. The biological plasticity of fetal life renders pregnancy a highly susceptible period for inducing positive effects mediated by lifestyle, particularly in high-risk situations. We hypothesized that lifestyle interventions focusing on improving nutrition and stress well-being could have an impact on improving fetal growth and perinatal outcomes in high-risk pregnancies.

### Objectives {7}

Our main hypothesis is that structured lifestyle interventions to improve nutrition based on MedDiet or to reduce stress with MBSR-based strategies will have an impact in improving fetal growth, perinatal outcomes, and infant development in high-risk pregnancies.

#### Primary objective

The primary objective is to evaluate the impact of the interventions (MedDiet and MBSR) on the rate of FGR at birth.

#### Secondary objectives

The following are the secondary objectives:

(i) To compare the effectiveness of the interventions (MedDiet and MBSR) in terms of reduction of adverse perinatal outcome (APO), defined according to the presence of any of the following perinatal measures: preterm birth, preeclampsia, perinatal mortality, severe FGR, metabolic acidosis, major neonatal morbidity
(ii) To evaluate the impact of the interventions (MedDiet and MBSR) on Bayley-III score at 2 years of corrected postnatal age of the infants
(iii) To evaluate the impact of the interventions (MedDiet and MBSR) on blood pressure and heart rate at 2 years of corrected postnatal age of the infants

### Trial design {8}

This is a RCT 1:1:1 ratio, parallel, open-blind. The study design adheres to the SPIRIT quality standard criteria for RCT [37].

## Methods: participants, interventions, and outcomes

### Study setting {9}

The study will take place in BCNatal (University Hospitals Clinic and Sant Joan de Déu, University of Barcelona), a large referral center for maternal-fetal and neonatal medicine in Spain, composed of two academic hospitals with more than 7,\000 deliveries/year and extensive experience in perinatal medicine.

### Eligibility criteria {10}

Eligible participants will be pregnant women at high risk to develop FGR during pregnancy according to the criteria of the Royal College of Obstetricians and Gynaecologists (RCOG) (odds ratio (OR) >2) [38].

The inclusion criteria are as follows:

- Age ≥18 years
- Speaking Spanish fluently
- Live singleton non-malformed fetus
- High-risk pregnancy to develop FGR (meeting the criteria of the RCOG)
- 19.0–23.0 weeks of gestation

The exclusion criteria are as follows:

- Fetal anomalies including chromosomal abnormalities or structural malformations detected by ultrasound prenatally and congenital infections
- Neonatal abnormalities diagnosed after birth
- Mental retardation or other mental or psychiatric disorders that impose doubts regarding the true patient’s willingness to participate in the study
- No possibility to come to additional visits
- Included in other RCT studies

### Who will take informed consent? {26a}

Doctors and researcher’s assistants involved in the trial will obtain written informed consent from potential trial participants.

### Additional consent provisions for collection and use of participant data and biological specimens {26b}

An additional written informed consent for biological sample collection will be obtained from the trial participants.

### Interventions

#### Explanation for the choice of comparators {6b}

The interventions are non-pharmacological, based on counseling and behavioral training, and derived from scientific evidence and tested in previous studies [18, 33]: (1) nutrition program based on MedDiet; (2) stress reduction program based on mindfulness techniques, MBSR; and (3) usual care without any intervention.

#### Intervention description {11a}

##### Nutrition program based on MedDiet

The dietary intervention is adapted from the PREDIMED (*Prevención con Dieta Mediterránea)* trial [18]. The main goal of the intervention strategy is to change general dietary patterns rather than focusing on changes in single food or macronutrients. The diet pattern will be adapted to pregnant women in general and will be further adapted after the first individual assessment visit with the dietician, according to the participant’s weight and culture. The intervention will be performed in individual 30-min visits plus a 1-h group session every month. Participants will also be provided with extra-virgin olive oil (2 l every month) and 15 g of walnuts per day (450 g every month) at no cost. Every 2 weeks, the woman will receive a phone call from the dietician in order to reinforce the intervention. Specific materials (recipes, a quantitative 1-week shopping list of food items according to the season of the year, a weekly plan of meals with detailed menus) will be given in each visit and will also be available in the Nutrition section (login with a password given at the moment of randomization) on the trial website (https://fetalmedbarcelona.org/impactbcn/). During each monthly visit, the 17-item dietary screener to assess baseline adherence to the MedDiet will be checked, and a 3-day dietary register will also be evaluated. In addition, blood pressure and weight gained will be measured.

##### Mindfulness-based stress reduction program

The program used for this trial is based on the MBSR program [39], which has been adopted by health institutions and tested in clinical trials [33, 40, 41], but it has been adapted to pregnant women, i.e., with meditations focused on the relationship with the fetus, “being with the baby” informal practice, and prenatal yoga positions. The intervention consists of 8 weekly sessions of 2.5 h plus one-full day session (20–25 people) and daily home practice. There is continuous monitoring/support by dedicated instructors to support practice and provide counseling throughout the 8 weeks and until delivery. Thus, after the program, extra weekly sections with meditations/yoga, sharing moments among women, and pregnancy-related problems will be offered by trained nurses to maintain adherence and practice. During sessions, mindfulness meditation skills are taught to help participants discover relationships between mindful practice and increase the ability to deal more effectively with stress. The sessions include didactic presentations, formal meditation practices, and group discussion. Home practice consists of 45’ of daily formal practice (e.g., sitting and walking meditation, body scan, and yoga stretching) and informal practice (e.g., mindfulness of daily activities and the 3-min breathing space). MP3s/CD of formal meditations adapted to pregnancy are provided for home practice, as well as a book and a notebook with relevant readings and the possibility to register their training. Extra material is also available on the official website of the trial (https://fetalmedbarcelona.org/impactbcn/) in the Mindfulness section (login with a password given at the moment of randomization).

#### Criteria for discontinuing or modifying allocated interventions {11b}

Not applicable due to the nature of the interventions.

#### Strategies to improve adherence to interventions {11c}

##### Participant compliance

Adherence to the nutritional intervention will be assessed by an improvement adherence to the MedDiet, based on an improvement of ≥3 points of their total final score of the 17-item dietary screener compared to their total initial score. The study population will be described according to adherence to the MedDiet, depending on the score: ≥12 high adherence, 6–11 moderate adherence, and <6 low adherence.

Adherence to the MBSR program will be assessed by class attendance and time spent engaged in mindfulness meditation outside of class sessions. The treatment will be considered complete if at least 10 h of meditation is done (both during class and at home), i.e., at least 6 sessions out of 9 are attended.

Biomarkers of compliance will be evaluated in a random sample of 30% of participants among the three arms of interventions, before and after the intervention: biomarkers of compliance with the nutrition program including (i) urinary total polyphenol excretion (adhesion to overall MedDiet pattern), (ii) urinary hydrohytyrosol concentration (compliance of extra-virgin oil), (iii) plasma alpha-linoleic acid concentration (compliance of walnuts), and (iv) biomarkers related to stress (steroid axis) will be evaluated by means of determination of cortisol in maternal 24-h urine [42].

##### Relevant concomitant care permitted or prohibited during the trial {11d}

Women who participated in this trial cannot participate in another trial.

#### Provisions for post-trial care {30}

Not applicable as no adverse event will be related to the trial due to the absence of any medication.

### Outcomes {12}

#### Primary outcome

The primary outcome will be the prevalence of FGR at birth, defined as newborns with a BW below the 10th centile [43] according to local standards [44].

#### Secondary outcomes

The following are the secondary outcomes:

(i) APO is defined as any of the following outcomes: (a) preterm birth, delivery <37 weeks’ gestation; (b) preeclampsia, defined as systolic blood pressure (SBP) ≥ 140 mmHg or diastolic blood pressure (DBP) ≥ 90mmHg at least 4 h apart after 20 weeks of gestation and proteinuria of ≥300 mg in 24 h; (c) perinatal mortality, fetal or neonatal mortality (within 28 days of life); (d) severe FGR, BW <3rd centile; (e) metabolic acidosis, an umbilical artery pH below 7.10 and/or base excess >12 mEq/L in the new-born and/or an Apgar score at 5 min below 7.0 assigned by the attending neonatologist or midwife; and (f) major neonatal morbidity in the presence of any of the following: intraventricular hemorrhage grade III/IV, necrotizing enterocolitis, periventricular leukomalacia, sepsis, bronchopulmonary dysplasia, and hypoxic-ischemic encephalopathy.
(ii) Bayley-III score at 2 years of corrected postnatal age will be evaluated by means of their four items score (raw and scaled): (a) cognitive, (b) language, (c) motor, and (d) socio-emotional.
(iii) The cardiovascular system at 2 years of corrected postnatal age will be evaluated by (a) SBP, (b) DBP, (c) mean arterial pressure, and (d) heart rate.

### Participant timeline {13}

**Fig. 1:**
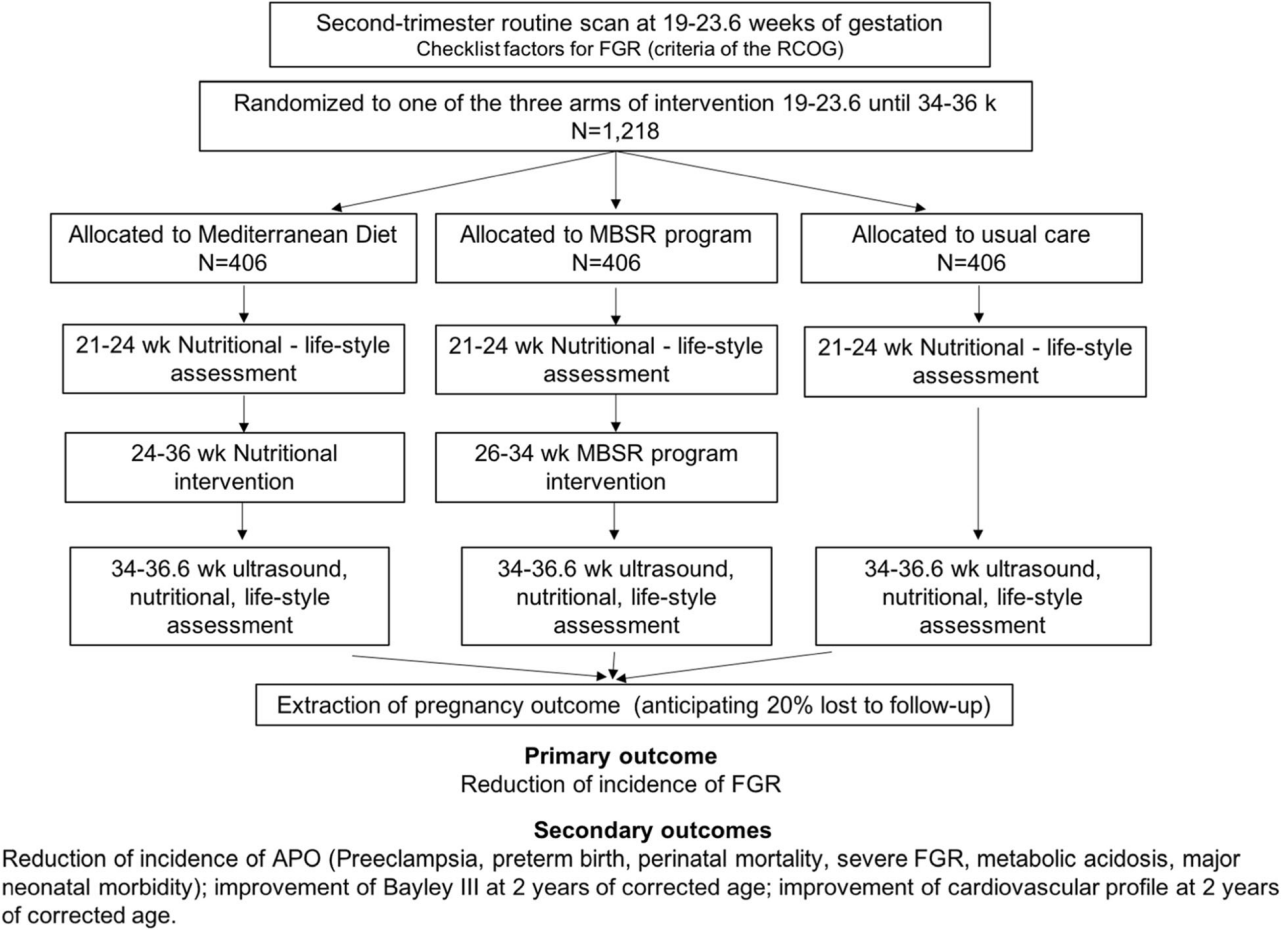
Flow chart of participants in the randomized controlled trial. FGR, fetal growth restriction; RCOG, Royal College of Obstetrics and Gynaecologists; MBSR, mindfulness-based stress reduction; APO, adverse perinatal outcome; PE, preeclampsia

Figure 1 shows the flow chart of participants, and Table 1 shows the study timeline.

**Table 1:** Study timeline

	Enrolment	Allocation	Post-allocation								Close-up
***Time point***	***Pregnancy***	***Pregnancy***	***Pregnancy***				***Delivery***	***Postpartum***			***Postpartum***
	*19–23.6 weeks*	*19–23.6 weeks*	*21–24.6 weeks*	*24–36 weeks*	*34–36.6 weeks*	*36–38 weeks*		*1–3 months*	*12 months*	*24 months*	*24 months*
	Screening visit	Randomization visit	Initial visit	Interventions	Final visit	Extra visit		Postnatal follow-up			
**Enrollment:**											
Eligibility screen	√										
Informed consent		√									
Allocation		√									
**Interventions:**											
MD				√							
MBSR program				√							
Usual care				√							
**Assessments:**											
Baseline variables											
Sociodemographic data			√								
Medical history			√		√						
Maternal life-style assessment			√		√			√		√	
Maternal nutritional assessment			√		√						
Outcome variables											
Primary outcome							√				
Perinatal outcomes							√	√			
Infant Bayley-III										√	√
Infant cardiovascular assessment										√	√
Other variables											
*Biological samples:*											
Maternal biological samples			√		√			√		√	
Feto-placental biological samples							√				
Infant biological samples								√		√	
*Maternal and fetal imaging:*											
Feto-placental US assessment					√						
EFW and feto-placental Doppler					√						
Fetal echocardiography					√						
Fetal neurosonography					√						
Maternal and fetal magnetic resonance rimaging						√					
*Infant extra tests:*											
Infant NBAS								√			
Infant ASQ									√		

*MedDiet* Mediterranean diet, *MBSR* mindfulness-based stress reduction, *US* ultrasound, *EFW* estimated fetal weight, *NBAS* Neonatal Behavioral Assessment Scale, *ASQ* Ages and Stages Questionnaires

### Sample size {14}

The study sample size (*n*=1218; 406 per arm) has been calculated assuming a type I error of 5% and aiming for a power of 80% for the most restrictive endpoint (reduction of 50% of the rate of APO). The sample size required for that secondary endpoint was actually more restrictive than the one required for the primary endpoint (reduction of 30% of the rate of neonates born with a BW <10th centile, *n*=1101; 367 per arm).

### Recruitment {15}

All women attending their routine second trimester ultrasound scan (19.0 23.6 weeks of gestation) will be invited to fill an eligibility questionnaire with a list of risk factors based on the criteria established by the RCOG [38]. The investigators involved in the recruitment will revise the inclusion/exclusion criteria and will address eligible patients about the purpose of the study and the voluntary nature of the participation. Women who agree to participate, after obtaining written informed consent, will be randomized and assigned a randomization code. Randomization will be performed using a web-based system which randomly assigns participants to one of the three different arms of intervention: (1) nutrition program based on MedDiet; (2) stress reduction program based on mindfulness techniques, MBSR; and (3) usual care without any intervention.

### Assignment of interventions: allocation

#### Sequence generation {16a}

The computer-generated randomization sequence is based on restricted randomization, with a forced strict number of participants per branch at the end of the trial. It is protected and managed by a computer technician of the foundation “*Medicina Fetal Barcelona*” which has no role in recruitment. The investigators enter data of the patient that is recruited on a website, and the computer generates automatically the inclusion number and treatment assignment. Participants are randomly assigned to either usual care, MedDiet, or MBSR with a 1:1:1 allocation as per computerized random number generator, with equal proportion in each group. An additional code is also generated, so the patient can have access to the official website of the study to a specific part according to her intervention (https://fetalmedbarcelona.org/impactbcn/).

#### Concealment mechanism {16b}

Participants are randomized using an online central randomization service. Allocation concealment is ensured, as the service does not release the randomization code until the patient has been recruited into the trial, which takes place after eligibility has been confirmed and the consent form signed.

The inclusion number of the patient and the treatment assignment will be printed and saved in the investigator’s files. A copy will be given to the patient.

#### Implementation {16c}

All participants who give consent for participation and who fulfill the inclusion criteria are enrolled and randomized. Randomization is requested by the investigator responsible for the recruitment. A different investigator, responsible for the allocation, generates the allocation sequence using an online central randomization service. The investigator responsible for the allocation finds out the study arm and gives the information about intervention allocation to the participant. The staff responsible for the recruitment is not allowed to receive information about the group allocation.

The enrollment of the participants and the follow-up of the trial will be done by doctors and research assistants of BCNatal.

The nutrition intervention will be conducted by two expert dieticians and supervised by the Research Group on Nutrition, Cardiovascular Disease and Aging from IDIBAPS - Hospital Clinic of Barcelona (Estruch R.), with huge experience in this field of research [18].

The mindfulness intervention based on the MBSR program will be coordinated by co-founders of the *Institute of Mindfulness*, with instructors certified by the Center of Mindfulness in Medicine, Health Care and Society, University of Massachusetts Medical School, Worcester, MA, USA. The program has been supervised by the head of the Psychiatry Service of Hospital Clinic de Barcelona (Vieta E.).

### Assignment of interventions: blinding

#### Who will be blinded {17a}

Due to the nature of the intervention, it is not possible to blind participants or researchers involved in the trial with respect to the study group.

#### Procedure for unblinding if needed {17b}

Not applicable, and the trial will be not blinded.

### Data collection and management

#### Plans for assessment and collection of outcomes {18a}

At the moment of enrollment (19.0–23.6 weeks of gestation), lifestyle questionnaires *(State-trait Anxiety Inventory-STAI* [45], *Perceived Stress Scale* [46], *WHO Five Well Being Index* [47], *Five Facet Mindfulness Questionnaire-FFMQ* [48], *Pittsburgh Sleep Quality Index-PSQI* [49]), a visit with a dietitian for a nutritional assessment (*Food Frequency Questionnaires-FFQ*, *MedDiet Adherence* without the inclusion of alcohol, *Minnesota Physical Activity*, all used in previous studies [18]), and biological samples (maternal hair, peripheral blood, urine, vaginal and fecal swaps) will be taken from each woman. Participants will be scheduled for a final visit with also a detailed ultrasound assessment (fetal biometry and Doppler, fetal echocardiography, fetal neurosonography) at the end of the interventions (34.0–36.6 weeks of gestation): the same lifestyle questionnaires, another visit with a dietitian for a nutritional assessment, and the same biological samples (maternal hair, peripheral blood, urine, vaginal and fecal swaps) will be taken again. Additionally, a subgroup of women will be randomly selected for a magnetic resonance of the maternal brain, fetal brain, and placenta. Maternal and feto-placental biological samples (cord blood and placenta) will be taken at birth. Perinatal outcomes will be recorded within 28 days after delivery. Postnatal follow-up consists of several visits at different ages of the assessment of neurological functions with neurodevelopmental tests, the assessment of cardiovascular functions (blood pressure, heart rate), and biological samples (fecal swaps for microbiota evaluation). Visits are scheduled at 1–3 months (*Neonatal Behavioral Assessment Scale-NBAS* [50]), at 12 months (*Ages and Stages Questionnaires-ASQ* [51]), and at 2 years of corrected age (*Bayley Third edition* [52]).

Maternal and fetal biological samples will be analyzed to assess compliance. Additional analysis will include biomarkers for maternal nutrition (vitamins, iron, folate, cholesterol, and lipid levels), metabolomics, epigenetics, and gut and vaginal microbiota.

#### Plans to promote participant retention and complete follow-up {18b}

Research nurses involved in the study will follow-up participants in order to improve their compliance and will conduct the postnatal follow-up.

#### Data management {19}

Participants’ data for this study will be anonymized and entered into an electronic case report form. Investigators will collect maternal sociodemographic and clinical data. The electronic records will be only accessible to members of the research team with limited access through the username and password of authorized personnel. Researchers who access this database will be responsible for the confidentiality of the data collected. Data will be accessed only for statistical processing at given intervals, by professional statisticians with no access to the personal information of the study subjects.

#### Confidentiality {27}

All medical information is recorded on the hospital server. No data will be externally available. All data of this trial will be anonymized.

#### Plans for collection, laboratory evaluation, and storage of biological specimens for genetic or molecular analysis in this trial/future use {33}

Biological samples will be processed after collection and stored in −80°C freezers at Hospital Clinic-IDIBAPS Biobank. All samples for specimen banking are stored in coded tubes without any other attached information that would allow identification of the individual from whom the sample is collected.

### Statistical methods

#### Statistical methods for primary and secondary outcomes {20a}

The statistical analysis will follow the general regulatory recommendations given in the ICHE9 Clinical Trials guidelines [53].

The inferential analysis for the main variable (proportion of newborns with a BW below the 10th centile) will be performed by means of OR using a logistic regression model, with a 95% IC. The method of Hochberg will be used for the adjusted *p* values for the comparison of each interventional arm vs. control, i.e., the result *p* values of two comparisons will be ordered descending; the following decisions about the positive results of the study will be considered:

- If the highest *p* value will be lowest than the threshold (0.0490), the study is positive for the two interventional arms vs. control.
- If the highest *p* value will be higher than the threshold (0.0490), the study is not positive for this, and the next variable *p* value should be lowest than the 0.0245 (0.0490/2) to be able to conclude the positive comparison of this interventional arm vs. control.

The specific analysis will be as follows:

1. Modified intention-to-treat population (mITT), defined as all randomized patients with neonates who did not end with a congenital malformation, diagnosed during pregnancy or in the postnatal period.
2. Population per-protocol (PP), defined as all subjects included in mITT that participated in at least one visit without protocol deviation that might impact the study’s main assessments. The protocol deviation considered in this study is the low compliance with treatments. The next compliance criteria will be a reason for exclusion:
  - MBSR group: the patient attends less than 6 of 9 sessions (<67%).
  - Nutrition group: the patient has a MedDiet score at final assessment <3 from the initial score.

The patient who withdrew informed consent will be excluded from all populations.

The main study analysis will be performed using the mITT, pre-randomization and baseline analyses, and primary and secondary efficacy analyses. The primary outcome analysis will also be performed using also ITT population as a supportive, PP population to test the robustness of the results.

No inferential analysis will be performed for the baseline comparability.

The SAS System3 (Release 9.4 or an upgraded version) or R version 3.5.2 (or upgrade version) will be used to analyze the datasets. Details for the statistical analyses are available in the statistical analysis plan, made by the external statisticians.

### Interim analyses {21b}

An interim analysis is planned at 50% of the study recruitment using the O’Brien-Fleming approach. The alpha-adjusted nominal level will be 0.0015 one-sided (0.0031 two-sided) at the interim look and 0.0245 one-sided (0.0490 two-sided) at the final analysis.

### Methods for additional analyses (e.g., subgroup analyses) {20b}

For the secondary analyses, no alpha adjusted will be made; all secondary statistical tests will be applied with a 0.05 two-sided significance level due to the exploratory purpose. All details are explained in the statistical analysis plan.

### Methods in analysis to handle protocol non-adherence and any statistical methods to handle missing data {20c}

The handling of missing data will follow the principles specified in the ICHE9 [53] and the CPMP/EWP/1776/99. Guideline on Missing Data in confirmatory trials [54].

Formal imputations will be only performed for the main variable (proportion of newborns with a BW <10th centile) where missing data will be imputed by the following two strategies:

- The main missing imputation strategy: the worst case will be imputed for all causes of missing data, i.e., BW < 10th centile.
- The secondary missing imputation strategy: multiple imputation with the observed rates in the control group in all cases.

The main analysis will be considered the worst-case imputation and the multiple imputation will be a sensitivity analysis.

For the remaining efficacy variables, no formal imputation will be performed, and the available data-only approach will be used. For the continuous efficacy variables, since the predefined approach mixed models for repeated measurements [55–57] is robust to assess the presence of missing at random, the analysis will be done with all subjects despite the presence of missing data. Of note, this method calculates the estimations based on the variance-covariance structure but without any formal imputations.

### Plans to give access to the full protocol, participant level-data, and statistical code {31c}

Statistical analysis plan and statistical code will be published.

### Oversight and monitoring

#### Composition of the coordinating center and trial steering committee {5d}

The coordinating center is BCNatal. The Trial Steering Committee is composed of principal investigators at BCNatal (Crovetto F, Crispi F, Gratacós E), responsible for each intervention (Estruch R for nutritional intervention, Vieta E for mindfulness intervention).

#### Composition of the data monitoring committee, its role, and reporting structure {21a}

An independent clinical trial unit will perform offline data auditing every 6 months to check for missing information and errors. The sites will be notified of any deviation from the standard recommendations for amendment. The Data Monitoring Committee (DMC) is independent from the trial researchers and is responsible for monitoring the progress of the trial including recruitment and protocol adherence and will also evaluate the results of the interim analysis, completely blind to any personnel external to the DMC.

### Adverse event reporting and harms {22}

As the trial will not use medical treatment, no adverse events are planned. However, principal investigators will collect any adverse event and will communicate it to the DMC.

### Frequency and plans for auditing trial conduct {23}

The DMC will give one to two reports during the trial. The processes reviewed related to participant eligibility, consent, and enrollment will periodically be revised by trained researcher members. All medical records related to the endpoints will be examined by trained and research staff members, blinded to the group assignment.

### Plans for communicating important protocol amendments to relevant parties (e.g., trial participants, ethical committees) {25}

Protocol modifications will be approved by the ethical committee of the hospital. The approval of the second amendment was on January 30, 2020.

### Ancillary and post-trial care {30}

We do not expect adverse events related to trial procedures given the non-pharmacological nature of the interventions. We plan and commit to provide care in our hospital for participants’ health care ancillary needs that may arise during trial participation (e.g., any maternal morbidity detected by the stress and well-being questionnaires, any fetal malformation detected by fetal US or magnetic resonance). Ours is a public hospital that can cover any maternal (at Departments of Psychiatry and Phycology, and Internal Medicine, Hospital Clínic), fetal (at the Maternal-Fetal Medicine Department, Hospital Clínic-Hospital Sant Joan de Déu), or infant (at the Neonatology Department, Hospital Clínic-Hospital Sant Joan de Déu) morbidity raised during the trial with no need to provide extra pay. We commit to provide ancillary care to the mother and fetus/infant during the trial duration (from recruitment until 2 years postpartum). We do not plan to provide care after the trial is completed.

### Dissemination plans {31a}

The results from this trial will be relevant for pregnant women, obstetricians, and healthcare professionals. The Steering Committee will review all publications. Trial results will be disseminated via (i) scientific publications through e-journals as well as high impact, international, peer-reviewed publications; (ii) scientific societies/conferences/scientific meetings; and (iii) the local, regional, and national press, television and radio outlets, and social media (Twitter, Linkedin, Facebook, Instagram, etc.).

## Discussion

Not applicable.

### Trial status

Protocol version 2, 23.10.2019. Recruitment began on February 1, 2017. Follow-up of the study will be completed in March 2022.

## Data Availability

The principal investigators (FCro, FC, and EG) and the statistic (RB) will have access to the final trial dataset, and they will not have any contractual agreement that limits such access.

## Abbreviations

APO: Adverse perinatal outcome
BW: Birth weight
DBP: Diastolic blood pressure
DMC: Data Monitoring Committee
FGR: Fetal growth restriction
ITT: Intention-to-treat
ITTm: Intention-to-treat modified
MBSR: Mindfulness-based stress reduction
MedDiet: Mediterranean diet
11β-HSD2: 11β-Hydroxysteroid dehydrogenase type 2
OR: Odds ratio
PP: Protocol analysis
RCOG: Royal College of Obstetricians and Gynaecologists
RCT: Randomized controlled trial
SBP: Systolic blood pressure
